# Minimally invasive exoscope-assisted coccygectomy: A novel approach for chronic refractory coccydynia

**DOI:** 10.1016/j.wnsx.2024.100412

**Published:** 2024-09-25

**Authors:** Barnabas Obeng-Gyasi, Danielle Wilmes, Matthew P. Blackwell, Jae H. Kwon, Gordon Mao

**Affiliations:** Department of Neurological Surgery, Indiana University School of Medicine, 355 W 16th St, GH #5100, Indianapolis, IN, 46202, USA

## Abstract

**Background:**

This technical note presents a novel minimally invasive exoscope assisted coccygectomy to treat chronic refractory coccydynia. Traditional treatments often fail to provide adequate relief for this debilitating condition, prompting the need to explore surgical approaches.

**Case Description:**

A 40-year-old female patient with persistent pain unresponsive to conservative treatments underwent this advanced procedure. Utilizing the Synaptive exoscope-microscope system, the surgery allowed for precise dissection and removal of affected coccygeal segments, with a focus on minimizing skin and soft tissue disruption to optimize wound healing and surgical site pain.

**Conclusion:**

Postoperative recovery showcased significant pain reduction and improved quality of life, emphasizing the method's potential for better outcomes and fewer complications. Despite the promising results, the limitations of a single-case study necessitate further research to establish long-term effectiveness across a broader patient population.

## 1 Introduction

Coccydynia, a debilitating condition causing pain in the coccyx, has been a recognized medical issue since the mid-19th century.^6^ The multifaceted etiology of coccydynia includes direct trauma, often from falls, biomechanical factors related to obesity and female gender, varying coccygeal morphology with certain anatomical configurations linked to higher pain incidence, abnormal coccygeal mobility, and discogenic or articular pain stemming from degenerative changes in the intercoccygeal articulation.^3^^,^^6^^,^^7^ Females, in particular, are more susceptible to coccydynia due to anatomical and physiological differences such as a straighter coccyx, which is more prone to injury by low-energy trauma, and the common occurrence of coccygeal trauma during childbirth.^13^

The complexity of this condition is magnified by the diverse anatomical configurations of the sacrococcygeal junction, each presenting varying risks for the development of coccydynia.^6^ Diagnostic imaging is critical for elucidating coccydynia's underlying causes, with standard lateral and anteroposterior radiographs serving as essential initial steps in the diagnostic process. Being a small, non-weight-bearing portion of the spine, the coccyx may commonly be overlooked in pelvic x-rays, contributing to the underdiagnosis of coccygeal pathologies. In cases of trauma or when standard imaging is inconclusive, dynamic imaging, and CT scans offer more detailed insights. MRI is also valuable, particularly for identifying signal alterations around the coccyx that may indicate the pain's origin.^12^

This paper explores the application of a novel minimally invasive exoscope-assisted coccygectomy for chronic refractory coccydynia. Through a detailed presentation of a technical note, we examine the surgical approach, technique, and patient outcomes to illuminate the potential of this procedure in the broader context of spinal surgery. The significance of this approach is discussed in terms of its contribution to reducing postoperative recovery times, minimizing complications, and improving patient quality of life. We also address the wider clinical implications, the lessons learned, and future directions for research.

## 2 Case presentation

### 2.1 Patient presentation

**Fig. 1:**
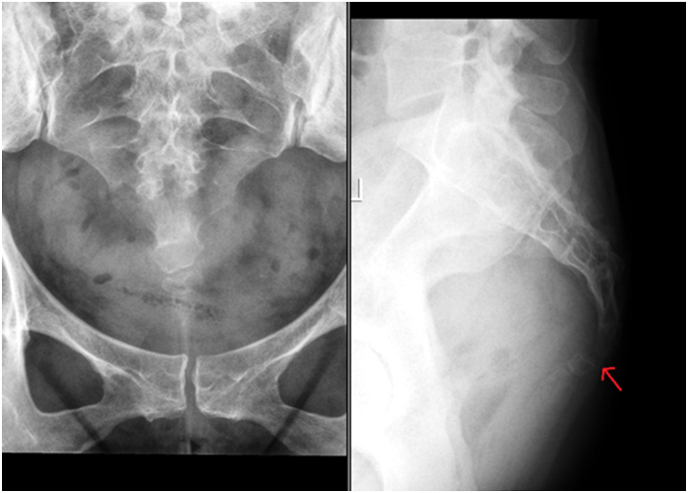
Preoperative coronal and sagittal x-rays demonstrating subluxed sacrococcygeal joint (red arrow).

A 40-year-old Caucasian female presented with a prolonged history of coccydynia that significantly impacted her quality of life. Initial management with steroid injections had offered temporary respite; however, their efficacy diminished over time, leading to a resurgence of symptoms. Review of the pelvic X-rays by the surgeon demonstrated a clear dislocation of the sacrococcygeal joint (Fig. 1). The deterioration in the patient's condition, coupled with the inadequacy of conservative measures, prompted the consideration of a minimally invasive surgical approach as a more definitive solution.

### 2.2 Surgical technique

The patient underwent surgery under general anesthesia. In the prone position on a Jackson table, she was carefully secured, ensuring the protection of pressure points. Utilizing a #10 blade and electrocautery, a midline incision was made, followed by insertion of the METRX retractor system, which facilitated a gradual dilation of the operative field to a 22 mm diameter. The strategic placement of the retractor, anchored by a jointed arm, provided an unobstructed view of the coccyx.

**Fig. 2:**
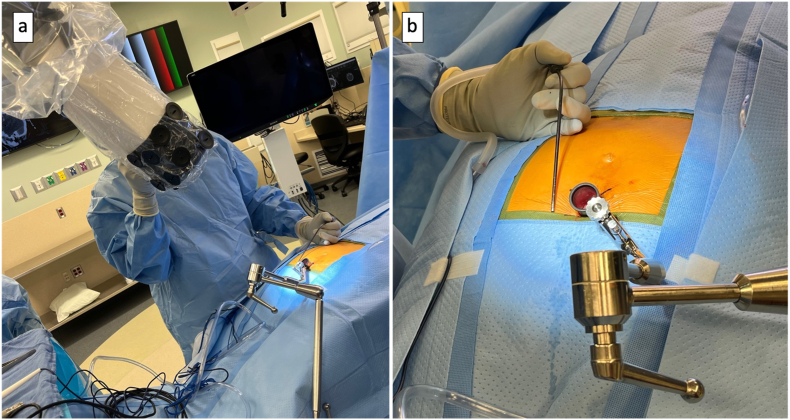
(a) Exoscope system setup. (b) View of surgical field for exoscope assisted coccygectomy using 22 mm diameter METRX retractors.

**Fig. 3:**
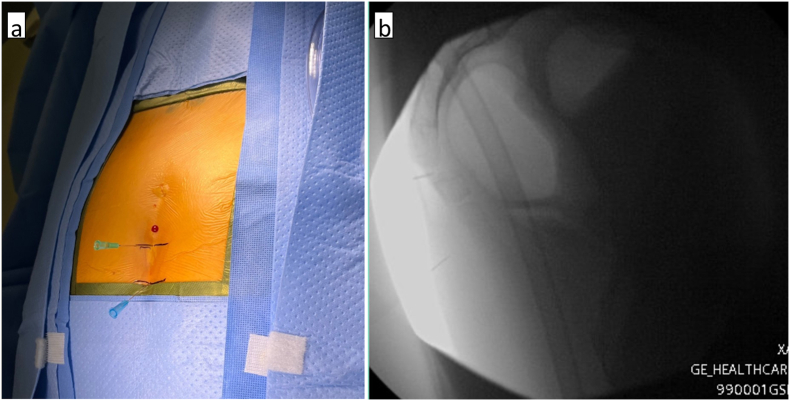
(a) Intraoperative localization with hypodermic needle. (b) Intraoperative localization with lateral fluoroscopic X-rays.

**Fig. 4:**
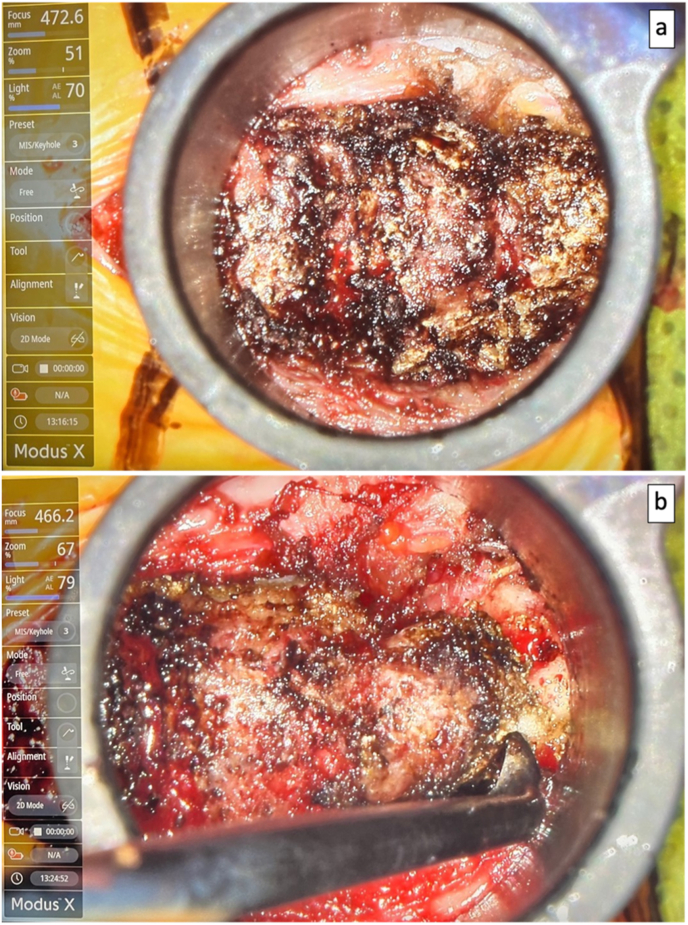
(a) Intraoperative view of coccyx through exoscope (b) Use of angled curette to dissect the coccyx en bloc from the surrounding myofascial attachments.

Employing the Synaptive exoscope-microscope system, microsurgical dissection was performed to meticulously expose the posterior elements of the S5, C1, C2, and C3 segments (Fig. 2). An intraoperative C-arm fluoroscopy confirmed correct anatomical localization (Fig. 3). Notably, pathological hypermobility was observed at the S5-C1 and C1-2 joints. A combination of low-power monopolar cautery and the use of angled curettes enabled the delicate separation of soft tissues and the en bloc removal of the C1, C2, and C3 segments (Fig. 4).

**Fig. 5:**
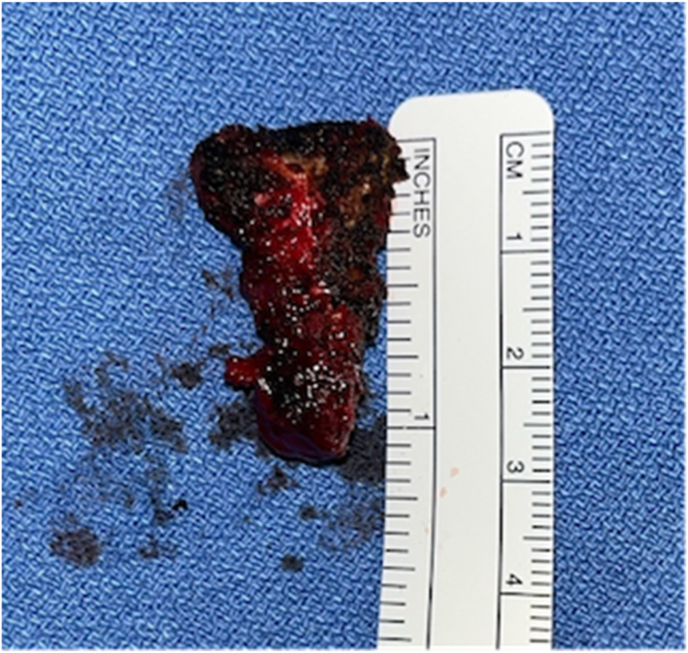
Resected coccyx.

Following the excision (Fig. 5), the surgical team conducted a thorough inspection of the retroperitoneal space and achieved thorough hemostasis. The closure of the wound was performed in a multilayered fashion—deep dermal layers and subcutaneous tissues were approximated using 2-0 Vicryl sutures, while the skin was closed with a continuous 3-0 Monocryl suture, bolstered by additional interrupted 3-0 Prolene sutures. The incision was dressed with Dermabond skin glue, concluding the procedure with an emphasis on structural integrity and infection prevention.

### 2.3 Postoperative care and outcome

The patient's postoperative course was carefully managed to ensure optimal recovery. Pain was managed with a multimodal analgesia regimen tailored to the patient's needs, which included both pharmacologic and non-pharmacologic strategies.

The patient was given comprehensive wound care instructions upon discharge, including how to monitor for signs of infection and guidelines on how to care for the surgical site. She was also instructed to avoid sitting on hard surfaces or remaining seated for prolonged periods of time to avoid excessive pressure on the incision and impairment of wound healing.

**Fig. 6:**
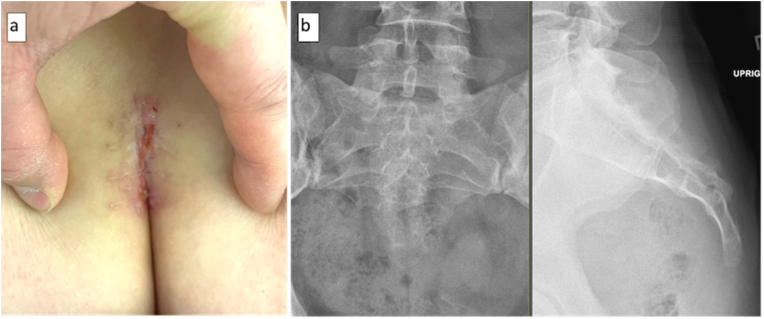
(a) Surgical site at four-week post-operative follow up. (b) Post-operative coronal and sagittal x-rays showing absence of the coccyx.

During follow-up, the patient reported a marked decrease in coccydynia. The wound exhibited satisfactory healing, characterized by well-approximated edges and the absence of erythema or exudate. She had minimal incisional pain and no signs of surgical site infection. Fig. 6 shows the wound at 4 weeks post-operation.

## 3 Discussion

Minimally invasive exoscope-assisted coccygectomy offers a promising glimpse into the future of spinal surgery.^10^ This technique stands on the shoulders of historical surgical methods while pushing the envelope of innovation to reduce patient morbidity associated with coccygectomy. Historically, the surgical management of coccydynia has seen a gradual but determined shift from open procedures to minimally invasive approaches/treatments, primarily to mitigate the high complication rates associated with the sacral area—a region prone to infection and wound breakdown due to its anatomical location and the high bacterial load.^5^^,^^11^ Rates of wound infection or delayed wound healing after coccygectomy have been reported to be anywhere between 2 and 30 %.^3^^,^^5^ One approach to minimizing risk of wound infection is minimally invasive surgery, as open spinal surgery has been shown to have a 5.77 times greater surgical site infection rate than minimally invasive spinal surgery.^2^

**Fig. 7:**
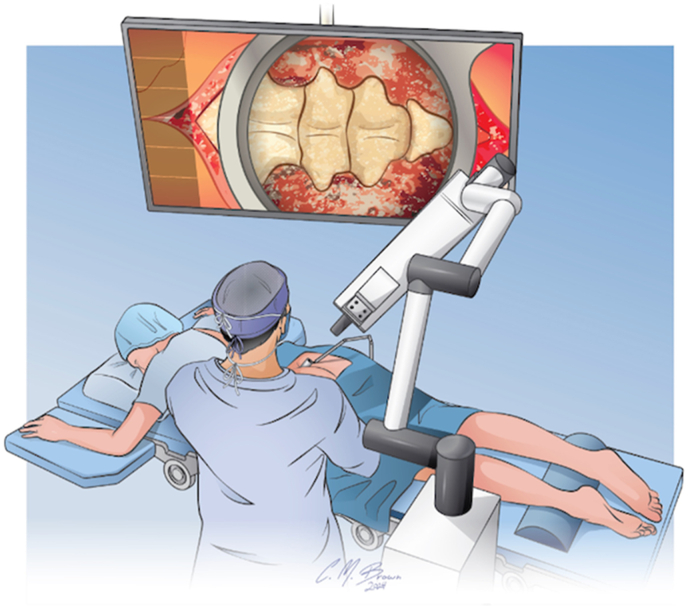
Illustrative depiction of intraoperative operating room setup for exoscope-assisted coccygectomy.

Our findings echo those in the literature that advocate for minimally invasive techniques due to their potential in minimizing tissue disruption and expediting patient recovery.^1^ The employment of an exoscope-assisted approach in our case has notably enhanced surgical precision—crucial for the intricate work required in spinal surgeries. Similarly to endoscopic technique, exoscopic procedures enhance surgeon ergonomics by providing the necessary view on a screen, eliminating the need to twist the neck to visualize the plane perpendicular to the coccyx. These devices also allow more precise visualization than would be possible with the naked eye or with the use of loupes. Unlike traditional endoscopic procedures, however, the exoscope provides a simplified setup without the need for an additional person to hold the endoscope, offering greater ease of operation and stability.^4^ The system we utilized confers other distinct advantages, including superior magnification, illumination, and 3D visualization as compared to standard endoscopy. These features facilitate a clearer and more detailed view of the surgical field, allowing for more precise dissections and potentially reducing the risk of complications. 3D visualization is especially relevant in order to clearly visualize and carefully dissect around the triangular, curved coccyx with intact depth perception to avoid injury to deep pelvic structures. Endoscopic surgery is traditionally touted in spine cases for requiring smaller incisions and allowing better visualization, as the scope is in close proximity to the spine and can provide views through the foramen or boney elements to better visualize the nerves. In coccygectomy cases, this is not relevant as there are no major nerves to visualize, dissect, or preserve. By incorporating exoscope technology, it is possible to reduce incisional pain, fascia and skin incision size, wound dehiscence, and surgical site infection rates compared to a traditional midline open approach (Fig. 7).

A unique consideration in our discussion is the integration of women's health perspectives. Women are disproportionately affected by coccydynia, experiencing condition 5x more often than men.^7^^,^^8^ This is often due to anatomical differences in the female pelvis that predispose them to injury, particularly during childbirth.^9^ The sparse literature available surrounding the screening, diagnosis, and treatment of coccydynia in women highlights the historical underrepresentation of women's health issues in medical research. By addressing these gaps, our case contributes to a more inclusive understanding of coccydynia and its management, advocating for gender-specific research within the field of spinal surgery.

The clinical implications of our findings are broad and significant. As neurosurgery continues to embrace minimally invasive techniques, our case points to the need for neurosurgical practices to evolve concurrently. The application of an exoscope-assisted approach may not only refine current practices but also pave the way for future technological integrations in spine surgery. With this case, we not only document a successful intervention but also invite a reconsideration of standard practices, suggesting that such innovative techniques could be more widely adopted, pending further validation through research and clinical trials.

## 4 Conclusion

This technical note illustrates the promising potential of minimally invasive exoscope-assisted coccygectomy for chronic refractory coccydynia. The procedure marks a significant step forward in spinal surgery, offering a glimpse into an advanced surgical approach that could lead to enhanced outcomes. The most salient lesson from this study is the effectiveness of employing minimally invasive techniques, especially exoscope-assisted methods, in managing complex spinal conditions. This case has shown that such an approach allows for precise and less disruptive surgical intervention, providing an additional option for patients whose conditions have not improved with standard treatment protocols.

However, it is important to recognize the limitations of this study. The long-term effects and generalizability of this technique remain uncertain, as the findings are based on a single case. Further research with a larger cohort is essential to establish the durability of these results and to determine whether they can be replicated across a diverse patient population.

In conclusion, while this case contributes valuable insights to the field of spinal surgery, it also underscores the necessity for ongoing research. The integration of this technique into broader clinical practice could indeed lead to significant improvements in the management of coccydynia and other similar conditions, but this potential can only be realized through continuous investigation and validation.

## CRediT authorship contribution statement

**Barnabas Obeng-Gyasi:** Conceptualization, Data curation, Investigation, Methodology, Validation, Visualization, Writing – original draft, Writing – review & editing. **Danielle Wilmes:** Conceptualization, Formal analysis, Writing – original draft, Writing – review & editing. **Matthew P. Blackwell:** Writing – original draft, Writing – review & editing. **Jae H. Kwon:** Data curation, Investigation, Writing – original draft, Writing – review & editing. **Gordon Mao:** Conceptualization, Data curation, Investigation, Project administration, Supervision, Writing – original draft, Writing – review & editing.

## Declaration of competing interest

The authors declare that they have no known competing financial interests or personal relationships that could have appeared to influence the work reported in this paper.

## Abbreviations:

S5: Fifth sacral vertebra
C1: First coccygeal segment
C2: Second coccygeal segment
C3: Third coccygeal segment
